# Prevalence of Nonclassic Congenital Adrenal Hyperplasia in Turkish Children Presenting with Premature Pubarche, Hirsutism, or Oligomenorrhoea

**DOI:** 10.1155/2014/768506

**Published:** 2014-03-23

**Authors:** Cigdem Binay, Enver Simsek, Oguz Cilingir, Zafer Yuksel, Ozden Kutlay, Sevilhan Artan

**Affiliations:** ^1^Division of Paediatric Endocrinology, Department of Paediatrics, Osmangazi University, School of Medicine, 26480 Eskisehir, Turkey; ^2^Department of Medical Genetics, Osmangazi University, School of Medicine, 26480 Eskisehir, Turkey

## Abstract

*Background*. Nonclassic congenital adrenal hyperplasia (NCAH), caused by mutations in the gene encoding 21-hydroxylase, is a common autosomal recessive disorder. In the present work, our aim was to determine the prevalence of NCAH presenting as premature pubarche (PP), hirsutism, or polycystic ovarian syndrome (PCOS) and to evaluate the molecular spectrum of *CYP21A2* mutations in NCAH patients. *Methods*. A total of 126 patients (122 females, 4 males) with PP, hirsutism, or PCOS were included in the present study. All patients underwent an ACTH stimulation test. NCAH was considered to be present when the stimulated 17-hydroxyprogesterone plasma level was >10 ng/mL. *Results*. Seventy-one of the 126 patients (56%) presented with PP, 29 (23%) with PCOS, and 26 (21%) with hirsutism. Six patients (4,7%) were diagnosed with NCAH based on mutational analysis. Four different mutations (Q318X, P30L, V281L, and P453S) were found in six NCAH patients. One patient with NCAH was a compound heterozygote for this mutation, and five were heterozygous. *Conclusion*. NCAH should be considered as a differential diagnosis in patients presenting with PP, hirsutism, and PCOS, especially in countries in which consanguineous marriages are prevalent.

## 1. Introduction

Nonclassic congenital adrenal hyperplasia (NCAH), caused by 21-hydroxylase deficiency, is one of the most common autosomal recessive disorders in humans, attributable to mutations in the* CYP21A2* gene and the related pseudogene* CYP21A1P*.

Genetic defects in the* CYP21A2* gene are classified into three categories depending on the extent of residual enzymatic activity. In vitro studies have shown that mutations causing complete inactivation of 21-hydroxylase activity are associated with the salt-wasting (SW) form of the condition, those that reduce 21-hydroxylase activity to about 2% of the normal value with the simple-virilising (SV) form of the disease, and those that reduce 21-hydroxylase activity to 10–75% of the normal value with the nonclassical (NC) form [1, 2].

The prevalence of NCAH varies among ethnic groups, and the condition has been estimated to occur in 0.1% of the general worldwide population but in 1-2% of Hispanics and Yugoslavs and 3-4% of Ashkenazi Jews [3]. Patients with NCAH exhibit variable clinical presentations, but all have signs and symptoms of excess androgen synthesis. Children and adolescents may present with premature pubarche (PP), hirsutism, polycystic ovarian syndrome (PCOS), menstrual dysfunction, severe cystic acne, male-pattern alopecia, advanced bone age, accelerated linear growth velocity, and decreased fertility [4].

PP is diagnosed based on exclusion of precocious puberty and NCAH. Most children with PP exhibit idiopathic premature adrenal androgen secretion. However, in 5–20% of such children, PP is caused by NCAH [5]. If it is desired to control the final height attained, early diagnosis is important and treatment should be commenced before signs of virilisation become obvious. At adolescence, females are more symptomatic than are males and may present with hirsutism, menstrual irregularity, or PCOS. These symptoms are thought to be caused by androgen excess impairing hypothalamic sensitivity to progesterone and in turn creating persistent GnRH pulsing and LH hypersecretion [4]. The gold standard test for diagnosis of NCAH is assay of stimulated adrenocorticotropic hormone (ACTH) level [6].

In the present study, we evaluated 126 Turkish children and adolescents with suspicious symptoms to determine the prevalence of NCAH. We assayed stimulated ACTH levels in patients presenting with PP, hirsutism, or PCOS and evaluated the molecular pattern of* CYP21A2* gene mutations in such patients.

## 2. Subjects and Methods

A total of 126 patients 3–17.8 years of age admitted to our Paediatric Endocrinology Department because of PP, PCOS, or hirsutism were studied. All patients underwent physical examination. Height was measured in the standing position, without shoes, using a stadiometer with a sensitivity of 0.1 cm. Weight was measured using a portable scale (sensitivity, 0.1 kg) with the patient dressed in light clothing. Body mass index (BMI) (weight (kg)/height (m)^2^) was recorded. Weight, height, and BMI standard deviations (SD) were calculated using reference curves for Turkish children [7, 8]. Pubertal status was evaluated using the criteria of Marshall and Tanner [9]. Bone-age SD was measured according to Greulich and Pyle [10]. Gender, chronological age, age at symptom onset, birth weight, weight SD, height SD, BMI SD, and bone-age SD were recorded, respectively. Small-for-gestational age (SGA) status was defined as a birth weight below the 10th percentile; children with birth weights between percentiles 10–90 were defined as appropriate-for-gestational age (AGA); and those with birth weights above the 90th percentile were considered to be large-for-gestational age (LGA) [11]. Precocious pubarche was defined as onset of pubic hair growth before the age of 8 years in females and 9 years in males [12]. Prospective subjects were excluded if they exhibited clinical signs of central precocious puberty. PCOS was diagnosed using the criteria of the National Institutes of Health (NIH), thus by clinical and biochemical evidence of hyperandrogenism and ovulatory dysfunction (menstrual irregularity) that could not be explained by the presence of another disorder [13]. The extent of hirsutism was evaluated in nine body areas using a modified Ferriman-Gallwey scoring system, and patients scoring eight or over on the scale were considered to be hirsute [14]. Idiopathic hirsutism was diagnosed in patients with hirsutism who had normal menstrual cycles, normal serum androgen concentrations, and no identifiable cause of hirsutism [15]. All patients underwent ACTH stimulation testing. Female adolescents were tested during the follicular phase of the menstrual cycle. After overnight fasting, 0.25 mg of ACTH (Synachten, Ciba-Geigy, Basel, Switzerland) was injected as an intravenous bolus between 08:00 a.m. and 10:00 a.m.; and cortisol, 17-hydroxyprogesterone (17-OHP), and dehydroepiandrosterone sulphate (DHEAS) levels were measured 0 (basal) and 60 min after ACTH administration. Cortisol and DHEAS levels were analysed using electrochemiluminescence assays (Roche E-170; Basel, Switzerland). 17-OHP levels were determined by ELISA. NCAH was considered present if the stimulated 17-OHP plasma level was greater than 10 ng/mL. Molecular analysis of the* CYP2A2* gene was performed on all patients diagnosed with NCAH based on the ACTH stimulation test.

## 3. Molecular Analysis of* CYP21A2*

Peripheral blood samples and genomic DNA from peripheral blood leukocytes were prepared using Roche MagNa Pure Compact Nucleic Acid Isolation Kits and Magna Pure Compact System Kits, respectively (Roche Diagnostics, Indianapolis, IN). Genetic analysis was conducted using a reverse-hybridisation strip-based assay (the CAH StripAssay) that explored the presence of the 11* CYP21A2* mutations most prevalent in European populations: P30L, IVS2 splice (IVS2 G), Del 8bp E3 (G110del8nt), I172N, Cluster E6 (I236N, V237E, and M239K), V281L, L307 frameshift (F306+T), Q318X, R356W, P453S, and R483P.

## 4. Statistical Analysis

SPSS for Windows 20.0 was used for analysis of raw data. The Shapiro-Wilk test was used to confirm that sample data were normally distributed. The parametric Independent Samples *t*-test was used to compare normally distributed data among groups. The nonparametric version of the Mann-Whitney *U*-test was used to perform comparisons among groups exhibiting nonnormal distribution of data. Pearson's chi-squared and Fisher's exact tests were used to conduct paired categorical data analysis. All data are expressed as means ± SD or median values obtained at percentiles 25 and 75 (Q1 and Q3). A *P* value <0.05 was considered to reflect statistical significance.

## 5. Results

Seventy-one of the 126 patients (56%) presented with PP, 29 (23%) with PCOS, and 26 (21%) with hirsutism. Mean age at symptom onset was 6.6 ± 1.2 years in PP patients, 12.6 ± 1.3 years in PCOS patients, and 11.5 ± 2.8 years in patients with hirsutism. Birth weight data were available for 108 patients; 18 children scored as SGA, four as LGA, and 86 as AGA. Ferriman-Gallway scores were available only for PCOS patients (mean: 19.7 ± 5) or those with hirsutism (mean: 20.6 ± 4). Clinical and biochemical data on all study subjects are shown in Table 1. Examination of 17-OHP values after ACTH stimulation revealed that nine patients (7.1%) had NCAH. Based on the genetic analysis, six patients (4.7%) were diagnosed with NCAH. The NCAH prevalence was 4.2% (*n* = 3) in PP patients, 6.8% (*n* = 2) in PCOS patients, and 3.8% (*n* = 1) in patients with hirsutism.

The clinical characteristics of and laboratory findings of all patients with or without NCAH are shown in Table 1. Basal and stimulated levels of 17-OHP and bone-age SD were significantly higher in those with than without NCAH (*P* < 0.05 for all comparisons).

Data on patients with or without NCAH were compared with those of PP patients. Nonpathological exaggerated secretion of androgen was defined as idiopathic PP (IPP). The clinical characteristics of and laboratory findings on IPP and NCAH patients are shown in Table 2. The stimulated levels of 17-OHP, and bone-age SD, were higher in children with NCAH (*P* < 0.05). However, no significant difference in any of chronological age, BMI SD, height SD, baseline or stimulated cortisol level, or baseline 17-OHP or DHEAS level was evident between children with PP and NCAH. Birth weight data were available for 60 patients; 13 children were of SGA status, two of LGA, and 45 of AGA. Birth weight did not differ between patients diagnosed with or without NCAH (*P* > 0.05).

The clinical characteristics of nine patients diagnosed with NCAH according to the ACTH stimulation test are shown in Table 3. Six of these patients presented with PP, two with PCOS, and one with hirsutism. Four different mutations (Q318X, P30L, V281L, and P453S) were found in six of the nine patients diagnosed with NCAH. One patient with PP had compound heterozygous mutations (V281L and P30L). Five patients were heterozygous for disease-related mutations. V281L and P453S mutations were detected in PP patients, P30L was detected in PCOS patients, and Q318X in patients with both PCOS and hirsutism. Consanguinity was in play in three of nine NCAH patients.

## 6. Discussion

Nonclassic congenital adrenal hyperplasia should be considered in the differential diagnosis of PP, PCOS, and IH, because neither clinical presentation nor androgen level is a reliable predictor of this disease. In the present study, we determined the prevalence of NCAH in children and adolescents presenting with symptoms of androgen excess and evaluated the clinical characteristics of such patients.

The prevalence of NCAH in children with PP varies between 0 and 40% among different populations [16–19], and the prevalence of NCAH causing hirsutism or PCOS is up to 20%, varying with both ethnicity and geographical area [20–22]. Few data have been gathered on the prevalence of NCAH in Turkish PP patients. Erdeve et al. [23] studied 159 patients, and Gonc et al. [24] reported 186 patients; the prevalences of non-classical CAH were 5.7% and 3.2%, respectively. In our cohort, six patients (4.7%) were diagnosed with NCAH based on the genetic analysis. The prevalence of NCAH was 4.2% (*n* = 3) in PP patients, 6.8% (*n* = 2) in PCOS patients, and 3.8% (*n* = 1) in patients with hirsutism. Unluhizarci et al. [25] conducted an extensive study of hirsute and hyperandrogenic females from various regions of Turkey. The cited authors concluded that NCAH was not prevalent in the Turkish population (the incidence was 2.1%) [25]. Akinci et al. [26] found that the prevalence of NCAH in hirsute adolescent females was 3.1%, and Yarman et al. [27] found an NCAH prevalence of 33% in Turkish females with hirsutism and PCOS; the data were confirmed by genotyping and HLA typing. The high prevalence noted in the cited report was considered to be associated with ethnic diversity in Istanbul. In another Turkish study, the prevalence of NCAH was 6.9% in Turkish woman with PCOS and 15% in those with IH [28].

The limitations of the present study include the small number of patients (three) diagnosed with NCAH causing hirsutism or PCOS; we were unable to compare adolescents with hirsutism to those with PCOS and NCAH. However, we have described the clinical and laboratory findings of patients exhibiting androgen excess and we compared data on all patients with those of nine patients with NCAH. We examined all IPP patients, and the six patients for whom NCAH was diagnosed as the cause of PP, in an effort to identify factors predictive of NCAH development. Most prior studies have proposed that patients with NCAH exhibit accelerated bone-age maturation [23, 29–31] and increased basal or stimulated levels of 17-OHP [29, 32, 33]. In the present study, as expected, the basal and stimulated levels of 17-OHP were higher (*P* < 0.001), and the bone-age SD was more advanced (*P* < 0.05), in children with NCAH. However, we found no between-group difference in any of current age, age at onset of symptoms, BMI, height, baseline and stimulated levels of cortisol, or DHEAS (*P* > 0.05 for all comparisons). Birth weight did not differ between patients diagnosed with or without NCAH (*P* > 0.05). Similarly, neither birth weight nor testosterone level was a predictor of NCAH development in the study of von Oettingen et al. [29], which involved 122 patients of varied ethnicity presenting with premature adrenarche. This result is in contrast with previous reports that elevated basal testosterone is a potential predictor of NCAH development [5, 33]. Ghizzoni et al. [34] studied the clinical and biochemical profiles of 152 Italian children with PP and genotypically screened all subjects. The cited authors could not define any diagnostic clinical characteristic of NCAH patients; even bone-age was not informative. Similarly, Ibáñez et al. [17] were unable to identify any clinical parameter differentiating PP patients into those with or without NCAH.

In our patient cohort, two of 29 PCOS patients and one of 26 patients with hirsutism were diagnosed with NCAH. Adolescent females with NCAH may suffer from gonadal dysfunction, menstrual disorders, or PCOS. These conditions are thought to be caused by conversion of adrenal androgens to oestrogens, altering the level of gonadotropin secretion or disrupting the cyclicity of gonadotropin release, thus directly affecting the ovary and ultimately leading to formation of androgen-producing cysts. Many authors have examined the similarity between NCAH and PCOS and the differences that might differentiate these two diseases. Obesity, insulin resistance, hirsutism, and polycystic ovarian morphology may be present in both NCAH and PCOS. The only exception is that the 17-OHP level is not elevated significantly in PCOS, at least not to the levels seen in NCAH. It has been suggested that NCAH should be excluded in patients presenting with hirsutism, oligomenorrhoea, and polycystic ovarian morphology. Alternations in* CYP21A2* gene transcription should be kept in mind as a cause of PCOS [22, 28, 35].

Molecular analyses of the* CYP21A2* gene were performed on nine patients diagnosed with NCAH according to the ACTH stimulation test. The V281L and P453S mutations were detected in PP patients; P30L was detected in PCOS patients; and Q318X was detected in patients with either or both of PCOS and hirsutism. Consanguinity was in play in three patients. Two PP patients in the V281L-heterozygous group had bone-age SD ≥2, and the basal levels of 17-OHP were 2.1 and 2.2 ng/mL, respectively. Consanguinity was in play in one patient. Both patients presented with hirsutism and PCOS were heterozygous for Q318X and had bone-age SD and peak 17-OHP levels of +2.3 and 10.8 ng/mL, respectively. The bone-age SD was <2 in a PCOS patient in whom P30L heterozygosity was recorded. In patients with relevant mutations, the maximum stimulated 17-OHP level was 29 ng/mL. It is surprising that the basal and peak levels of 17-OHP were higher in patients in whom we did not detect any mutation. This may indicate that it is important to evaluate other regions of the* CYP21A2* gene to detect rare mutations causing disease.

Previous studies have shown that mutations exerting mild phenotypic effects (the V281L, P30L, and P453S mutations) result in retention of 20–50% of enzymatic activity, and mutations exerting severe phenotypic effects (I2 splice, I172N, and Q318X) 0–2% [3, 36–38]. An association is evident between genotype and phenotype. Homozygous mutations exerting mild phenotypic effects and heterozygous mutations with mild or severe phenotypic effects trigger the nonclassical form of the disease [38]. Our results are consistent with these earlier data.

The phenotypic heterogeneity of NCAH patients can be explained by the simultaneous expression of different mutations. Such patients may carry an unidentified mutation in the other* CYP21A2* allele or may carry a mutation exerting a mild phenotypic effect in one allele only [3]. This suggests that patients who are compound heterozygotes for one mutation exerting a mild phenotypic effect and another exerting a severe phenotypic effect may be younger have a greater height SD, exhibit a more advanced bone age, and express a higher 17-OHP level at presentation [2, 36]. However, in the absence of full sequences of the* CYP21* gene and regulatory elements thereof, we cannot presently draw such conclusions. The V281L mutation was more common than the P30L mutation in NCAH patients presenting with PCOS and hirsutism [21, 39, 40]. As found previously, the Q318X mutation was also noted in patients presenting with PCOS [22, 41]. Erdeve et al. [23] detected the V281L mutation in all PP patients of a Turkish population and suggested that the condition was caused by this mutation rather than other known mutations. Moreover, it has been reported that the V281L mutation is the most common mutation in Turkish patients with PP, PCOS, and hirsutism [25, 27]. In the present study, the Q318X, P30L, and P453S mutations were detected, in addition to the V281L mutation, in four patients presenting with PP, PCOS, or hirsutism. The P453S mutation was previously noted in Turkish children with NCAH [42, 43]. In other countries, the P453S and P30L mutations were found in 23.1% and 10.3%, respectively, of NCAH patients [3]. Helmberg et al. [38] identified R339H and P453S mutations associated with NCAH and proposed that each mutation caused a 50% reduction in normal* CYP21A2* activity. In another study, P453S was found in 46.2% of NCAH patients [44].

In conclusion, NCAH is a heterogeneous disorder in terms of age of onset, symptomology, and causative mutation. NCAH should be a differential diagnosis in patients presenting with PP, hirsutism, PCOS, and/or advanced bone age, especially in countries in which consanguineous marriages are prevalent. Investigating the molecular genetic mechanism of NCAH should resolve the diagnostic difficulties encountered with hormone testing. Genotyping should be considered for defining biological cohorts and it should shed light on whether the genetic variation is related to the disease in question. Of our nine NCAH patients, five had heterozygous and one had compound heterozygous mutations. Other disease-related mutations may also be present in such patients. Therefore, sequencing is the gold standard for identification of disease-associated mutations.

## Figures and Tables

**Table 1:** Clinical characteristics and laboratory findings of study subjects.

	Subjects without NCAH	Subjects with NCAH	*P* value
Age (year)	8,80 (7,30–14,60)	7,80 (6,80–13,30)	0,336
Gender (female/male)	113/4	9/0	0,741
BMI SDS	0,82 ± 0,09	1,03 ± 0,36	0,572
Height SDS	0,33 ± 1,10	0,49 ± 0,39	0,685
Bone Age SDS	1,02 ± 0,09	2,07 ± 0,12	**0,002**
Ferriman Gallway Score	20,18 ± 4,65	23,00 ± 5,00	0,594
T.Testosterone (ng/dL)	16,50 (0,69–38,37)	7,05 (0,79–20)	0,752
Cortisol_0_ (*μ*g/dL)	14,41 ± 5,72	13,75 ± 5,68	0,740
Cortisol_1 hour_ (*μ*g/dL)	28,69 ± 5,66	32,47 ± 5,30	0,055
17-OHP_0_ (ng/mL)	1,20 (0,90–2)	3,90 (2,15–9,50)	**<0,001**
17-OHP_1 hour_ (ng/mL)	3,10 (2,25–4,40)	21,60 (9,80–34,05)	**<0,001**
DHEA-S_0_ (*μ*g/dL)	103 (53,50–181,50)	128 (128–331,50)	0,619
DHEA-S_1 hour_ (µg/dL)	102 (53,70–188,50)	130 (50,50–312,50)	0,622

Data are expressed as the means ± standard deviations or medians (Q1–Q3) as appropriate. NCAH: nonclassic congenital adrenal hyperplasia; BMI-SDS: body mass index standard deviation score; 17-OHP: 17-hydroxyprogesterone; DHEA-S: dehydroepiandrosterone sulphate.

**Table 2:** Comparisons of clinical and laboratory findings between patients with nonclassic congenital adrenal hyperplasia and premature pubarche.

	IPP (*n* = 65)	NCAH (*n* = 6)	*P* value
Gender (female/male)	61/4	6/0	0,697
Age (year)	7,50 (6,95–8,20)	6,90 (6,60–8,85)	0,400
BMI SDS	0,85 ± 0,99	0,94 ± 1,17	0,827
Height SDS	0,71 ± 1,11	0,91 ± 1,15	0,676
Bone Age SDS	1,12 ± 1,02	2,11 ± 0,43	**0,022**
T.Testosterone (ng/dL)	9,47 (0,30–20)	20 (4,82–45,50)	0,258
Cortisol_0 hour_ (*μ*g/dL)	13 ± 5,36	11,86 ± 3,14	0,594
Cortisol_1 hour_ (*μ*g/dL)	29,20 (24,30–32,20)	31,70 (29–35)	0,121
17-OHP_0 hour_ (ng/mL)	1,18 ± 1,01	6,33 ± 4,96	0,052
17-OHP_1 hour_ (ng/mL)	3 (2,20–4)	25,30 (9,97–39,32)	**<0,001**
DHEA-S_0 hour_ (*μ*g/dL)	65,20 (43,70–107)	62,40 (40,77–128,50)	0,960
DHEA-S_1 hour_ (*μ*g/dL)	67,70 (43,45–101)	64,50 (45,70–131,25)	0,848

Data are expressed as means ± standard deviations or medians (Q1–Q3) as appropriate. IPP: idiopathic premature pubarche; NCAH: nonclassic congenital adrenal hyperplasia; BMI SDS: body mass index standard deviation score; 17-OHP: 17-hydroxyprogesterone; DHEA-S: dehydroepiandrosterone sulphate.

**Table 3:** Clinical characteristics and genotypes of patients with nonclassic congenital adrenal hyperplasia.

Patient	Genotype	Clinical feature	Age	Gender	Consanguinity	BA-SDS	17-OHP_0_	17-OHP_peak_
1	NA	PP	6,8	F	No	+1,5	13	40
2	V281L/P30L	PP	6,0	F	No	+2,6	2,1	10
3	NA	PP	7,8	F	No	+1,7	10,6	39
4	NA	PP	6,8	F	No	+2,4	8,4	21,6
5	Q318X	Hirsutism	13,1	F	Yes	+2,3	2,3	10,5
6	Q318X	PCOS	13,5	F	No	+2,0	3,9	10,8
7	P30L	PCOS	15,9	F	Yes	+1,7	8,1	26,5
8	V281L	PP	7,0	F	Yes	+2,4	2,2	29
9	P453S	PP	12,0	F	No	+2,1	1,7	10

NA: not applicable; BA-SDS: bone age-standard deviation score; 17-OHP: 17-hydroxyprogesterone; PP: premature pubarche; PCOS: polycystic ovarian syndrome.
